# Calcium Influx Caused by ER Stress Inducers Enhances Oncolytic Adenovirus Enadenotucirev Replication and Killing through PKCα Activation

**DOI:** 10.1016/j.omto.2019.09.003

**Published:** 2019-09-28

**Authors:** William K. Taverner, Egon J. Jacobus, John Christianson, Brian Champion, Adrienne W. Paton, James C. Paton, Weiheng Su, Ryan Cawood, Len W. Seymour, Janet Lei-Rossmann

**Affiliations:** 1 Department of Oncology, University of Oxford, Roosevelt Drive, Oxford OX3 7DQ, UK; 2 NDORMS, Botnar Research Centre, University of Oxford, Headington, Oxford OX3 7LD, UK; 3 PsiOxus Therapeutics, Ltd., Milton Park, Abingdon OX14 3YS, UK; 4 Research Centre for Infectious Diseases, Department of Molecular and Biomedical Science, University of Adelaide, Adelaide SA 5005, Australia; 5 Oxford Genetics Ltd., Medawar Centre, Robert Robinson Avenue, Oxford OX4 4HG, UK

**Keywords:** adenovirus, calcium, PKC, oncolytic, ER stress

## Abstract

Oncolytic viruses represent an emerging approach to cancer therapy. However, better understanding of their interaction with the host cancer cell and approaches to enhance their efficacy are needed. Here, we investigate the effect of chemically induced endoplasmic reticulum (ER) stress on the activity of the chimeric group B adenovirus Enadenotucirev, its closely related parental virus Ad11p, and the archetypal group C oncolytic adenovirus Ad5. We show that treatment of colorectal and ovarian cancer cell lines with thapsigargin or ionomycin caused an influx of Ca^2+^, leading to an upregulation in E1A transcript and protein levels. Increased E1A protein levels, in turn, increased levels of expression of the E2B viral DNA polymerase, genome replication, late viral protein expression, infectious virus particle production, and cell killing during Enadenotucirev and Ad11p, but not Ad5, infection. This effect was not due to the induction of ER stress, but rather the influx of extracellular Ca^2+^ and consequent increase in protein kinase C activity. These results underscore the importance of Ca^2+^ homeostasis during adenoviral infection, indicate a signaling pathway between protein kinase C and E1A, and raise the possibility of using Ca^2+^ flux-modulating agents in the manufacture and potentiation of oncolytic virotherapies.

## Graphical Abstract

**Figure:**
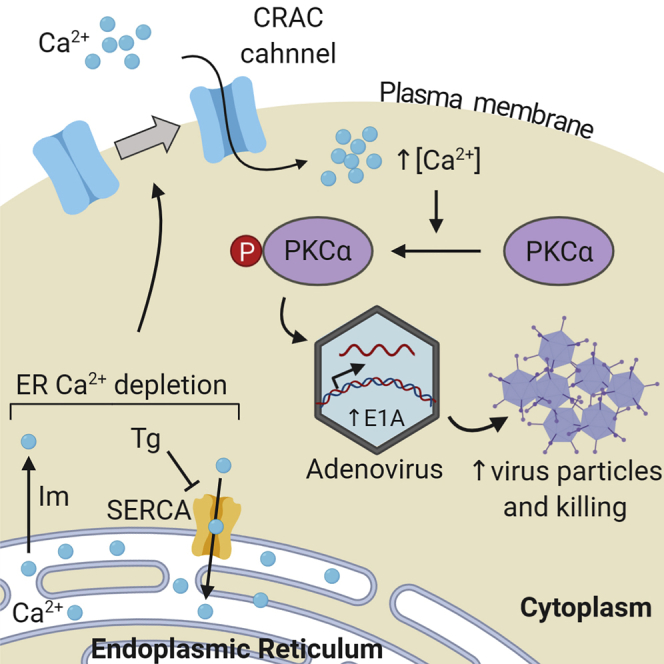

## Introduction

There is increasing interest in the deployment of oncolytic viruses (OVs) in the treatment of cancer, many offering powerful cancer-killing properties.^1^ In contrast to conventional treatments, OVs exploit the overall cancer phenotype to achieve specificity, benefiting from a unique ability to self amplify upon entry into tumor cells.^2^ Their infectious cycle culminates in the lysis of the host cancer cell, releasing progeny virus particles that establish subsequent rounds of infection and cell death in neighboring cancer cells. Lysis of infected cells can also contribute toward an inflammatory and immune response in the tumor, further enhancing cell killing.^3^, ^4^, ^5^ OVs may also be engineered to express immune-modulatory transgenes within tumor cells, allowing for a high local concentration of an immunotherapeutic in the cancer microenvironment and avoiding off-target toxicities.^6^, ^7^, ^8^ Combination strategies have also been explored as a means to potentiate further the activity of OVs.

Tumor cells must successfully synthesize and fold new proteins in order to ensure their survival and continued growth.^9^ When protein-folding homeostasis is disrupted, unfolded protein accumulates within the lumen of the endoplasmic reticulum (ER), a state known as ER stress. In response to this stress, the cell activates the concomitant restorative signaling response, known as the unfolded protein response (UPR).^10^ ER stress can be induced by many cellular insults.^11^ In cancer cells, these include their intrinsic genetic instability and aberrant protein production, as well as extrinsic conditions, such as hypoxia and nutrient depravation. In many cases, tumor progression is dependent on cellular adaptation to ER stress by induction of UPR signaling.^12^ Viral infections can also induce ER stress due to the substantial burden they impart on protein production and folding.^11^^,^^13^, ^14^, ^15^, ^16^ Many viral infections also subvert the UPR, manipulating the signaling response to benefit their own replication.^17^ The ability to sensitize cancer cells to OV-induced oncolysis by treating the cells with chemicals and biological agents to induce cellular stress is virus dependent. Some viruses have shown enhanced replication and cell killing upon induction of stress,^18^, ^19^, ^20^, ^21^ whereas others displayed attenuated activity.^22^

Adenoviruses are nonenveloped double-stranded DNA viruses. The adenovirus serotype that has historically been used as a model for adenoviral research and is most commonly used as both a vector in gene therapy and as an oncolytic agent is the group C adenovirus serotype 5 (Ad5).^23^ Within the fields of oncolytic virotherapy and gene therapy, adenoviruses are particularly widely used^24^ for a variety of reasons, including their high stability, large capacity for encoding transgenes, and ease of production. Seroprevalence of neutralizing antibodies against Ad5 is high,^25^^,^^26^ representing an important obstacle to their clinical application.^27^ In contrast, Enadenotucirev (EnAd) is an oncolytic adenovirus isolated through a process of directed evolution in HT-29 colorectal cancer cells and benefits from low seroprevalence.^28^ The EnAd genome is a chimera of group B adenovirus serotypes 3 and 11p and shows selective cancer cell killing in cocultures with normal cells *in vitro*,^29^ as well as an encouraging targeting and safety profile in an early clinical trial.^30^ EnAd can be used as a vector for the efficient and cancer-selective expression of immune-regulatory biologics^7^^,^^8^ and can reach the tumor after being administered systemically into humans.^5^^,^^30^, ^31^, ^32^

Although little is known about the interaction of ER stress and the UPR with adenoviral infection, it has been shown that treatment of cells with golgicide A, a chemical inhibitor of guanine nucleotide exchange factor-1 that triggers the UPR, enhances tumor cell killing by Ad5 infection.^18^ Here, we report that treatment of cancer cells with chemicals, widely used as ER stress inducers, thapsigargin (Tg) and ionomycin (Im), enhances the oncolytic activity of group B oncolytic adenoviruses EnAd and Ad11p. This enhanced killing was not a product of enhanced UPR activation but rather as a consequence of altered Ca^2+^ flux. We find that these chemicals accelerate the onset of replication and oncolytic killing by enhancing early viral gene expression through protein kinase C (PKC) signaling.

## Results

### Treatment of Cancer Cell Lines with Tg and Im Significantly Enhances Viral Protein Production

We examined the effect of three common ER stress inducers, Tg, Im, and tunicamycin (Tm), on the rate of viral protein production following infection of either SK-OV-3 ovarian or DLD-1 colorectal cancer cells with EnAd or Ad5. Tg irreversibly inhibits the sarco/ER Ca^2+^-ATPase (SERCA) channels, leading to a depletion of Ca^2+^ within the ER. Im is an ionophore that facilitates the movement of Ca^2+^ across membranes. Tm blocks N-linked glycosylation in the ER. To monitor viral protein production throughout the virus replication cycle, we used viruses engineered to express a reporter protein, GFP, under control of either the constitutive cytomegalovirus immediate-early promoter (CMV) promoter (EnAd-CMV-GFP and Ad5-CMV-GFP), which express GFP immediately upon entry of the genome into the nucleus, or the adenoviral major late promoter via a splice acceptor site (EnAd-SA-GFP), which controls expression of adenoviral late genes.^33^

**Figure 1:**
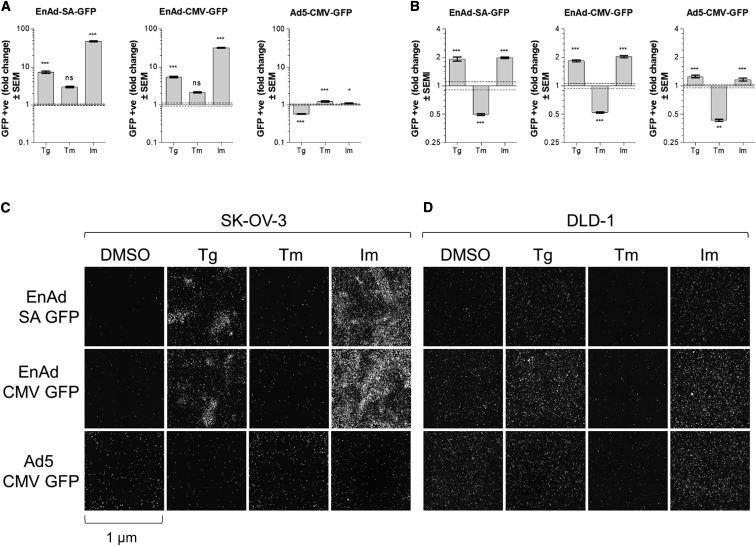
Effect of ER Stress-Inducing Chemicals on EnAd and Ad5 Transgene Expression SK-OV-3 ovarian cancer cells (A) or DLD-1 colorectal cancer cells (B) were plated in a 96-well plate, infected with EnAd-SA-GFP, EnAd-CMV-GFP, or Ad5-CMV-GFP and treated with Tg (0.1 μM), Im (1.25 μM), or Tm (1 μg/mL). Cells were imaged on a Celigo image cytometer and GFP expression quantified at 72 h (A) or 24 h (B) postinfection. Data represent average fold change in the percentage of GFP-positive cells from 60 replicates relative to DMSO vehicle control-treated cells; error bars represent ± SEM. Broken line represents ± SEM of the DMSO control. Significance was evaluated using one-way ANOVA. *p ≤ 0.05; **p ≤ 0.01; ***p ≤ 0.001; ns, nonsignificant. (C) Representative images of virus-encoded GFP expression for the conditions in (A). Image fields represent 1 μm. (D) Representative images of virus-encoded GFP expression for the conditions in (B).

In SK-OV-3 ovarian cancer cells, Tg and Im treatment drastically increased the percentage of cells expressing EnAd-encoded GFP, as measured 3 days post-infection (p.i.). EnAd reporter expression increased by approximately 7- and 47-fold with Tg and Im treatment, respectively, whereas Tm treatment did not result in a significant change compared to the untreated control. This effect was observed in viruses encoding for GFP under the control of either CMV or the major late promoter. During Ad5-CMV-GFP infection, the percentage of cells expressing GFP did not change substantially, with fold changes of 0.5, 1.2, and 1.1 for Tg, Im, and Tm, respectively (Figure 1A). In DLD-1 cells, a very similar pattern of effects was observed, although the fold changes were more modest, likely reflecting the greater permissivity of DLD-1 cells to adenoviral infection. The proportion of GFP-positive cells upon EnAd-SA-GFP and EnAd-CMV-GFP infections doubled following treatment with Tg or Im, whereas treatment with Tm caused a 0.5-fold reduction in GFP expression. CMV-driven GFP expression from a replicating Ad5 reporter virus (Ad5-CMV-GFP) did not change when cells were treated with Tg or Im (1.25- and 1.16-fold, respectively), whereas Tm treatment reduced GFP expression by approximately one-half (Figure 1B). At the selected time points, no secondary infection occurred in untreated cells. However, in treated cells, acceleration of the viral replication cycle led to more rapid lysis of cells infected during primary infection and therefore, subsequent rounds of infection. This can be seen visually by the increased number of infection “comets,” which are indicative of secondary infection and spread to neighboring cells,^34^ in Tg- and Im-treated but not untreated SK-OV-3 and DLD-1 cells (Figure 1C).

### Tg Accelerates Onset of Group B Oncolytic Adenovirus Replication, Infectious Particle Production, and Cell Killing

**Figure 2:**
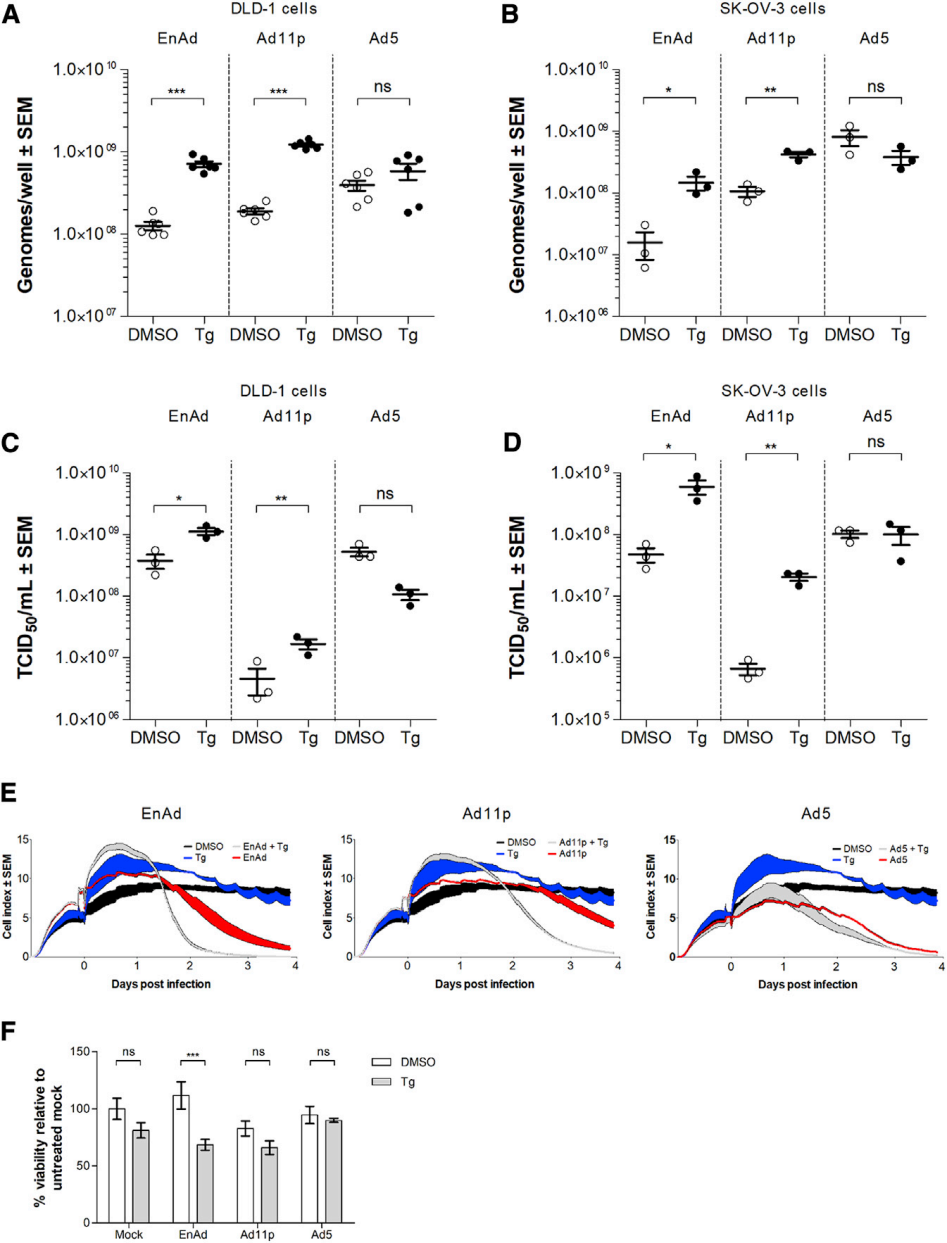
Thapsigargin Treatment Enhances Parameters of Infection of EnAd and Ad11p but Not of Ad5 (A and B) qPCR measurement of viral genomes in DLD-1 cells 24 h postinfection (p.i.) (A) and in SK-OV-3 cells at 72 h p.i. (B) in the presence or absence of Tg (0.1 μM) or vehicle control DMSO. (C and D) TCID_50_/mL of infectious virus particles in DLD-1 cells 24 h p.i. (C) and SK-OV-3 cells 72 h p.i. (D). Error bars represent ± SEM. Significance was evaluated by two-tailed t test. *p ≤ 0.05; **p ≤ 0.01; ***p ≤ 0.001; ns, nonsignificant. (E) Virus killing of DLD-1 cells was monitored by xCELLigence in the presence or absence of Tg (0.1 μM) after virus infection. Impedance was measured every 10 minutes. Data show the mean of four replicates with the weight of the line spanning ± SEM. An infectious dose of 100 VPC was used in all experiments. (F) Virus-induced cell cytotoxicity was assessed by MTS. DLD-1 cells were infected with EnAd, Ad11p, or Ad5. Cell viability was measured at 48 h p.i. Mean of six replicates is displayed per treatment group; error bars represent ± SEM. Significance was tested by two-way ANOVA. ***p ≤ 0.001; ns, non-significant.

The permissivity of SK-OV-3 cells and DLD-1 cells to infection by EnAd, Ad11p, and Ad5 was investigated in normal and Tg-treated conditions. Viral genomes were quantified by qPCR in the presence and absence of Tg treatment. In DLD-1 cells at 24 h p.i., Tg treatment increased EnAd genome replication by 9-fold and Ad11p genome replication by 4-fold, whereas wild-type Ad5 genome replication levels remained unchanged (Figure 2A). Tg treatment of SK-OV-3 cells resulted in a 6-fold increase in EnAd and Ad11p genomes at 72 h p.i. but no significant difference in Ad5 genomes (Figure 2B).

To measure whether the enhancements to viral protein and genome production translated into increased production of infectious progeny virions, SK-OV-3 or DLD-1 cells were infected with EnAd, Ad11p, or Ad5, and the number of infectious particles was quantified at 24 h and 72 h p.i., respectively. Upon Tg treatment, the number of EnAd particles increased 3-fold and Ad11p particles 4-fold; the number of Ad5 particles remained unchanged with treatment (Figure 2C). The number of EnAd and Ad11p particles released from SK-OV-3 cells increased 14- and 30-fold, respectively, with Tg treatment, whereas no significant difference was observed with Ad5 infection (Figure 2D).

We then evaluated whether treatment of DLD-1 cells with Tg sensitized them to virus killing using xCELLigence, which allows a real-time, label-free recording of cell impedance, measured as the cell index (CI). CI is a function of cell morphological parameters, such as cell adhesion, cytotoxicity, and proliferation. Monitoring changes in such a measurement over time provides a useful means to track virus-induced cytotoxicity and cell killing. Very little to no cytotoxicity was observed in uninfected DLD-1 cells treated with Tg (Figure 2E). Meanwhile, untreated cells infected with EnAd, Ad11p, or Ad5 exhibited signs of cytotoxicity, approximately 36 h p.i. Tg treatment promoted earlier cell killing by EnAd and Ad11p, at around 24 h p.i., indicated by the more vertiginous decline in CI and the earlier time point at which a CI of 0 is reached; this effect was absent in Ad5-infected cells. These results were supported by a 3-(4,5-dimethylthiazol-2-yl)-5-(3-carboxymethoxyphenyl)-2-(4-sulfophenyl)-2H-tetrazolium, inner salt (MTS) viability assay in Tg-treated infected cells, with EnAd showing significantly enhanced cell killing in the presence of Tg at 48 h p.i. (Figure 2F).

### EnAd E1A Expression Is Enhanced in Tg-Treated Cells

**Figure 3:**
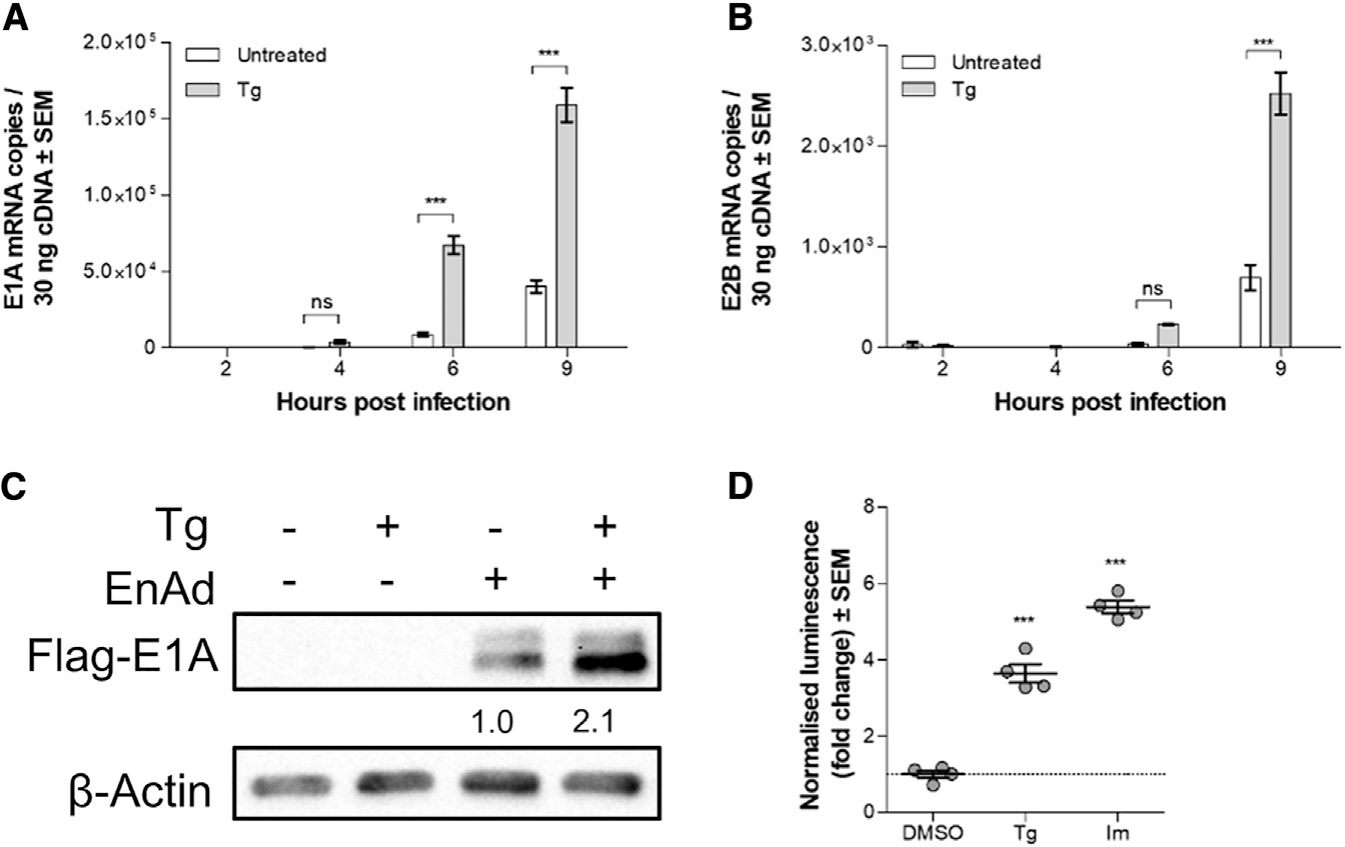
Thapsigargin Treatment Enhances Early Viral Gene mRNA and Protein Levels (A and B) Quantification of EnAd mRNA levels of E1A (A) and E2B (B) by qRT-PCR. Data represent the mean of three biological replicates, each an average of three technical replicates. Significance was assessed by two-way ANOVA using Bonferroni post-test. ***p ≤ 0.001; ns, non-significant. Error bars represent ± SEM. (C) Western blot of FLAG-E1A protein levels in DLD-1 cells 12 h postinfection with EnAd-FLAG-E1A (MOI, 3). Numbers indicate average normalized fold upregulation relative to DMSO control of three independent replicate experiments. (D) DLD-1 cells were infected with Ad5-E1A-Luc (MOI, 3) and treated with Tg (0.1 μM) or Im (1.25 μM). Cells were lysed to measure luciferase expression 12 h post-infection. Significance was assessed by one-way ANOVA. ***p ≤ 0.001; ns, non-significant. Error bars represent ± SEM.

We quantified transcript levels of the immediate-early adenoviral gene E1A and the early gene E2B by qRT-PCR following infection of DLD-1 cells. E1A is the first viral gene to be expressed upon infection and regulates the transcription of other early viral genes, including E2B, which is the viral DNA polymerase responsible for replication of the viral genome. We found a significant upregulation in the number of E1A transcripts upon Tg treatment at 6 and 9 h p.i. (Figure 3A). This enhancement was accompanied by the expected concomitant increase in E2B transcript levels (Figure 3B). To verify whether the observed enhancement in E1A mRNA levels was also present at the protein level, we used EnAd-FLAG-E1A (E.J.J. , unpublished data), an EnAd variant engineered to include a FLAG-tag on the E1A protein, to allow its detection by western blotting. In DLD-1 cells, we found that Tg treatment led to a 2-fold increase in FLAG-E1A signal level (Figure 3C; Figure S1). Infection of DLD-1 cells with Ad5-E1A-Luc,^35^ an Ad5 variant with luciferase fused to the E1A protein, showed that E1A-Luc levels were also significantly enhanced by Tg and Im treatments (Figure 3D).

### UPR Induction Is Not Responsible for the Accelerated Onset of Viral Replication

**Figure 4:**
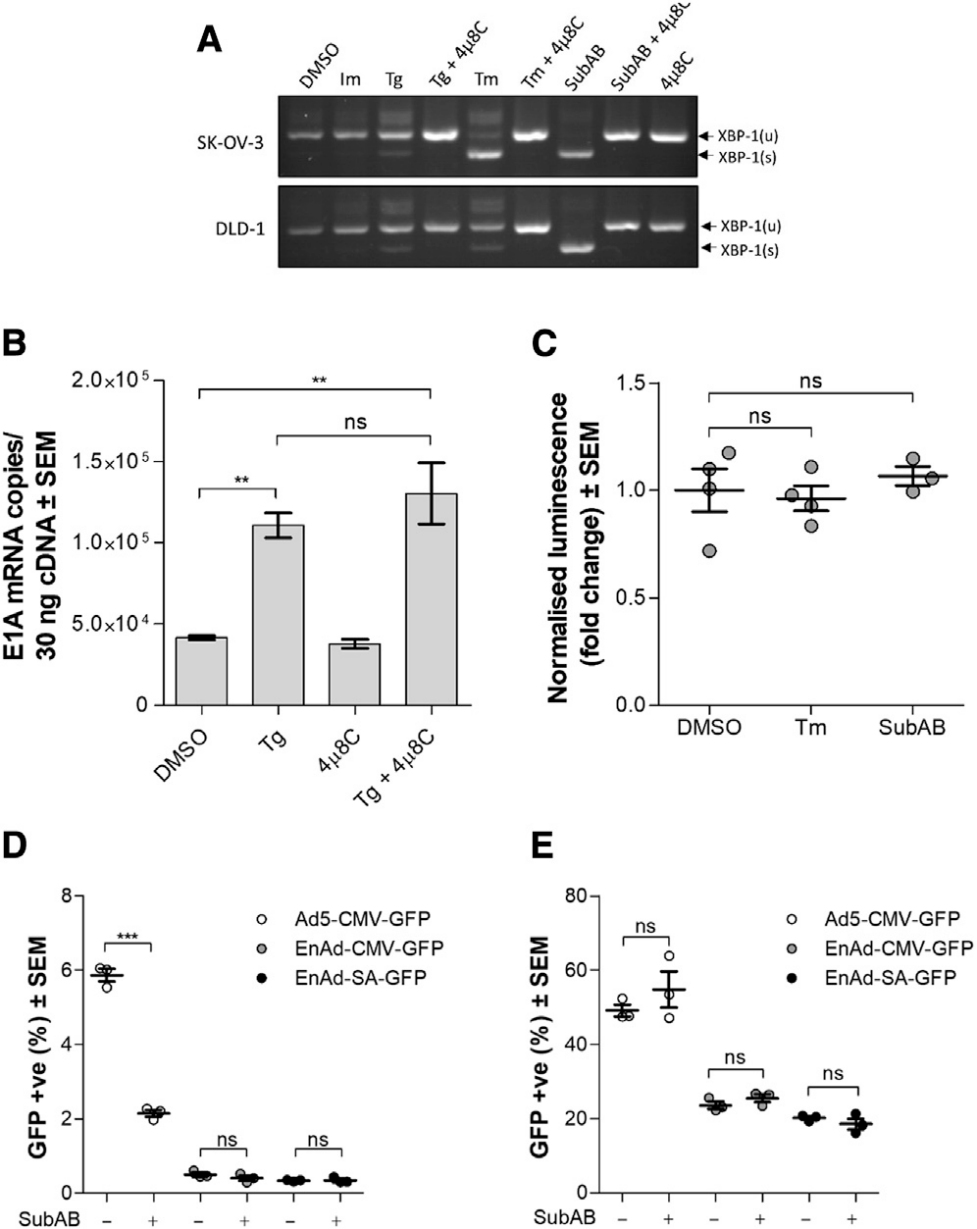
UPR Induction Is Not Responsible for the Increase in Early Viral Gene Expression or Virus Activity (A) XBP-1 splice status in SK-OV-3 or DLD-1 cells treated with the indicated chemical for 12 h. XBP-1(u) was observed at 283 bp; XBP-1(s), 257 bp. (B) qRT-PCR quantification of EnAd E1A mRNA in DLD-1 cells 9 h postinfection. The mean of three replicates is plotted. (C) Quantification of E1A-Luc reporter protein levels in DLD-1 cells 12 h post-infection with Ad5-E1A-Luc. (D and E) SK-OV-3 (D) and DLD-1 (E) were infected with EnAd-CMV-GFP, EnAd-SA-GFP, or Ad5-CMV-GFP and subsequently treated with SubAB. Data show the percentage of GFP-positive cells 72 h (D) and 24 h (E) postinfection, as quantified by Celigo. Significance was assessed by one-way ANOVA. ***p ≤ 0.001; **p ≤ 0.01; ns, non-significant. The following concentrations were used: Tg, 0.1 μM; Tm, 1 μg/mL; SubAB, 0.5 μg/mL; and 4μ8C, 100 μM. Error bars indicate ± SEM.

We originally hypothesized that treatment with chemical ER stressors enhanced E1A mRNA levels via downstream signaling molecules activated upon UPR induction. The most highly conserved signaling arm of the UPR is the inositol-requiring enzyme 1 (IRE1) signaling pathway. Upon induction following ER stress, the endonuclease activity of IRE1 is activated, allowing it to degrade many ER-targeted mRNAs. Among its targets is the X-box binding protein 1 (XBP-1) transcript, IRE1 directs the splicing of the unspliced XBP-1 transcript (XBP-1(u)), excising a 26-bp intron to yield the spliced transcript (XBP-1(s)). To assess the extent to which the treatments in our experimental system were leading to UPR activation, we measured the levels of the spliced versus unspliced XPB-1 transcripts in cells. In this experiment, we included treatment with the subtilase cytotoxin (SubAB). SubAB acts in a highly targeted manner by specifically cleaving BiP/GRP78.^36^ BiP/GRP78 is an ER resident chaperone protein that binds to and inhibits the activation of the ER stress-sensing signal transducers (protein kinase RNA-like ER kinase [PERK], activating transcription factor 6 [ATF6], and IRE1). The release of the ER stress-sensing molecules from the inhibitory binding of BiP in the ER lumen allows the rapid and potent induction of UPR signaling. We found that the Tg and Im treatments induced low levels of XBP-1 splicing, whereas Tm and SubAB were much more powerful in eliciting the UPR in both SK-OV-3 and DLD-1 cells. Inclusion of 4μ8C, an IRE1 inhibitor, abolished XBP-1 splicing as expected (Figure 4A).

We tested whether the increase in E1A mRNA copies observed following Tg treatment was dependent on IRE1 activation by including 4μ8C in the treatment media. Inhibition of IRE1 failed to ablate the enhancement to E1A transcript levels induced by Tg treatment (Figure 4B). The effects of 4μ8C and inhibitors of the other two UPR signaling arms (ATF6 and PERK) on the activity of EnAd-SA-GFP were also measured (Figure S2). IRE1 inhibition by 4μ8C was strongly prejudicial to virus activity but did not ablate the enhancing effect of Tg treatment. Ceapin-A7 was used to inhibit ATF6^37^ and had no effect on virus activity in the presence or absence of Tg. PERK inhibition by GSK2656157 had no effect on virus activity, whereas Tg was still able to mediate a significant, albeit reduced, increase in virus activity.

To measure E1A levels during Ad5 infection, cells were infected with Ad5-E1A-Luc. No change in luciferase signal was observed when DLD-1 cells were treated with Tm or SubAB (Figure 4C). SubAB treatment of SK-OV-3 cells decreased GFP expression after infection with Ad5-CMV-GFP but not EnAd-CMV-GFP or EnAd-SA-GFP (Figure 4D). Treatment of DLD-1 with SubAB after infection with EnAd-CMV-GFP, EnAd-SA-GFP, or Ad5-CMV-GFP had no effect on the percentage of GFP-positive cells (Figure 4E).

Together, these results show that the agents that induced UPR signaling most strongly (Tm and SubAB) were also the agents with the smallest effects on adenoviral activity (Figures 1A, 1B, 4D, and 4E). Meanwhile, Tg and Im, which induced UPR signaling more modestly, consistently showed the greatest enhancing effect to virus activity. These results highlight a reverse correlation between the level of UPR induction and enhancement of adenovirus activity.

### Influx of Extracellular Ca^2+^ into the Cytoplasm Enhances EnAd Activity

**Figure 5:**
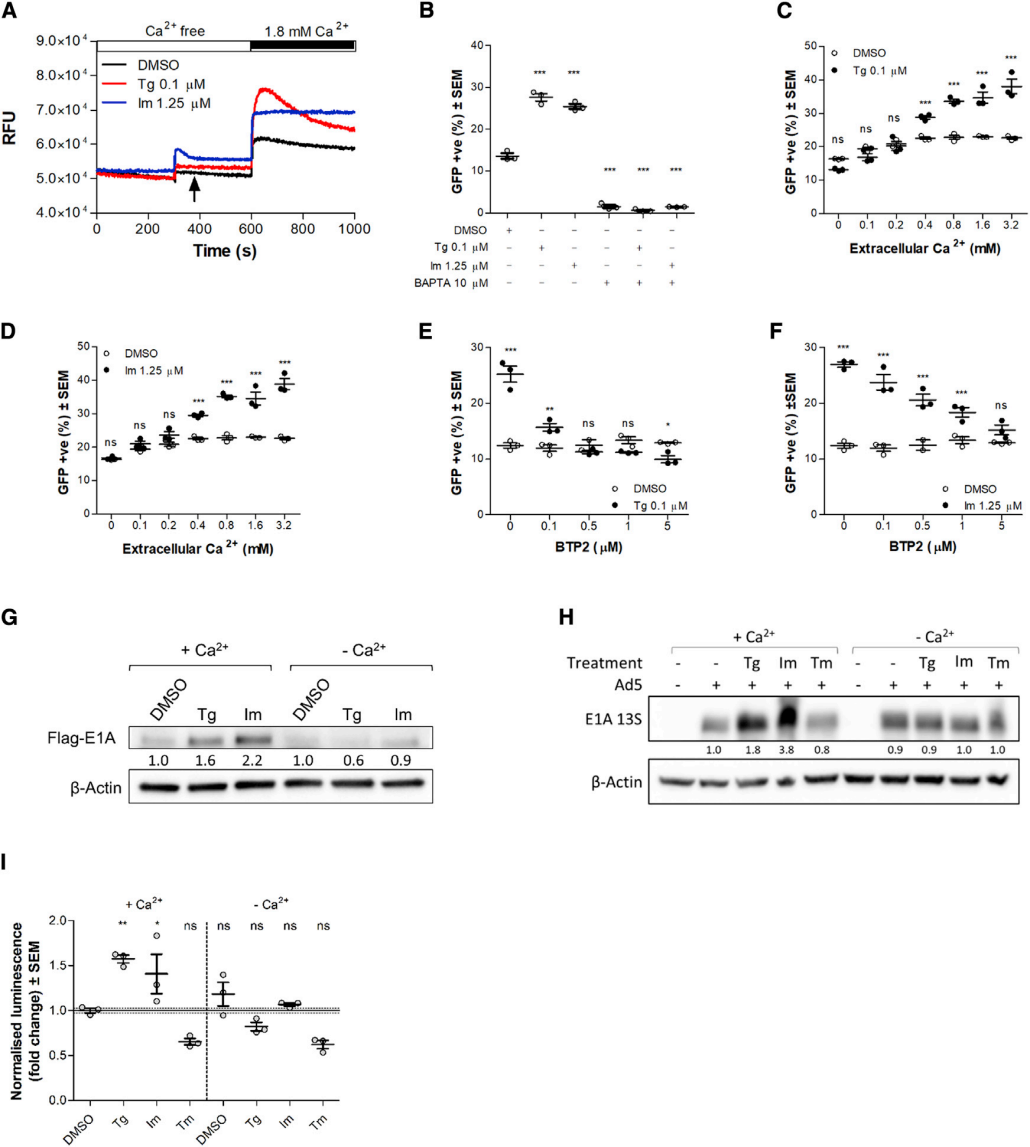
Influx of Extracellular Calcium Mediates Enhancement to Virus Activity Seen upon Treatment of DLD-1 Cells with Tg and Im (A) Measurement of cytosolic Ca^2+^ levels of DLD-1 cells over time. Drug treatments were injected into wells at 5 min, and Ca^2+^ was injected back into the media after 10 min. One representative experiment is shown. RFU, relative fluorescence units. (B) DLD-1 cells were infected with EnAd-SA-GFP in the presence of 10 μM BAPTA and subsequently exposed to Tg, Im, or vehicle control DMSO. Cells were imaged by Celigo 24 h postinfection. (C and D) DLD-1 cells were infected with EnAd-SA-GFP and subsequently treated with Tg (C), Im (D), or vehicle control DMSO. Treatment media contained variable concentrations of Ca^2+^ and were supplemented with 2% dialyzed (Ca^2+^ free) FCS. Cells were imaged by Celigo 24 h postinfection. (E and F) DLD-1 cells were infected with EnAd-SA-GFP in the presence or absence of BTP2. Cells were subsequently exposed to Tg (E) or Im (F) and imaged by Celigo 24 h postinfection. (G and H) Western blot of E1A levels in DLD-1 cells 12 h postinfection with (G) EnAd-FLAG-E1A (MOI, 3) or (H) wild-type Ad5 (100 VPC) and treated with ER stress inducers in the presence or absence of extracellular Ca^2+^. Numbers represent fold change relative to DMSO control within the relevant Ca^2+^ environment. (I) E1A-Luc levels were measured in DLD-1 cells 12 h postinfection with Ad5-E1A-Luc. Cells were treated with ER stress inducers in the presence and absence of extracellular calcium. Significance of differences in GFP expression levels relative to the DMSO control group were assessed by one-way ANOVA (B and I) or two-way ANOVA (C–F). ***p ≤ 0.001; **p ≤ 0.01; *p ≤ 0.05; ns, nonsignificant. Error bars represent ± SEM.

Since activation of the UPR signaling did not explain the observed enhancement to virus activity, we considered other explanations. Tg and Im both alter Ca^2+^ flux into the cell. We proceeded to investigate how such changes in Ca^2+^ flux might affect virus infection. With the use of a Ca^2+^-sensitive dye, we verified that Tg and Im treatments lead to increased cytosolic levels of Ca^2+^. In the absence of extracellular Ca^2+^, both treatments induced a small increase in cytosolic Ca^2+^ concentration, although the effect of Im was greater (Figure 5A). Five minutes after treatment, when Ca^2+^ was added back into the extracellular medium, a substantial increase in the cytosolic Ca^2+^ level was seen in all groups. However, more highly elevated levels were observed in cells that had been treated with Tg or Im compared to DMSO-treated cells. These elevated levels remained higher for the duration of the experiment (Figure 5A).

We found that depletion of free Ca^2+^ in the infection medium by chelation with 1,2-bis(o-aminophenoxy)ethane-*N*,*N*,*N*′,*N*′-tetraacetic acid (BAPTA) severely compromised infection. No enhancement of virus activity was observed when BAPTA-treated cells were concurrently treated with either Tg or Im (Figure 5B). These results agree with previous findings that extracellular calcium is required for viral uptake and penetration.^38^ Since we had found that treatment with both Tg and Im elevated cytosolic Ca^2+^ levels and that the source of this Ca^2+^ was predominantly extracellular, we sought to investigate the impact of removing extracellular Ca^2+^ after initial virus uptake on the enhancing effect of Tg treatment to EnAd activity. Initial infections were carried out in complete culture media with normal levels of free calcium. We found that the increase in EnAd activity observed upon Tg or Im treatment in the presence of normal levels of Ca^2+^ decreased in a dose-dependent manner as the Ca^2+^ concentration was reduced (Figures 5C and 5D).

Depletion of the ER Ca^2+^ store has been shown to cause opening of the Ca^2+^ release-activated Ca^2+^ (CRAC) channels in the plasma membrane, leading to increased levels of Ca^2+^ uptake from the extracellular environment.^39^, ^40^, ^41^ We reasoned that both Im and Tg treatments would induce an increase in cytosolic Ca^2+^ levels by activating the CRAC channels and that this could be the relevant factor driving EnAd activity enhancement. We found that increasing dosages of BTP2, a potent inhibitor of plasma membrane CRAC channels, reduced the enhancing effect of Tg and Im treatment in a dose-dependent manner (Figures 5E and 5F). Western blotting revealed that upregulation of EnAd E1A levels upon Tg or Im treatment was dependent on the presence of extracellular Ca^2+^ (Figure 5G). Changes in the levels of Ad5 E1A expression were also investigated (Figure 5H). In agreement with Figure 3D, western blotting showed that E1A protein levels also increased in response to Tg and Im treatment and that these increases were dependent on the presence of extracellular calcium. Tm, on the other hand, did not enhance Ad5 E1A levels. These results were further corroborated by the observation that DLD-1 cells infected with Ad5-E1A-Luc expressed more luciferase when cells were treated with Tg or Im, but not Tm, and that these increases were also dependent on extracellular calcium (Figure 5I).

### PKC Activation Mediates Enhanced EnAd Activity

**Figure 6:**
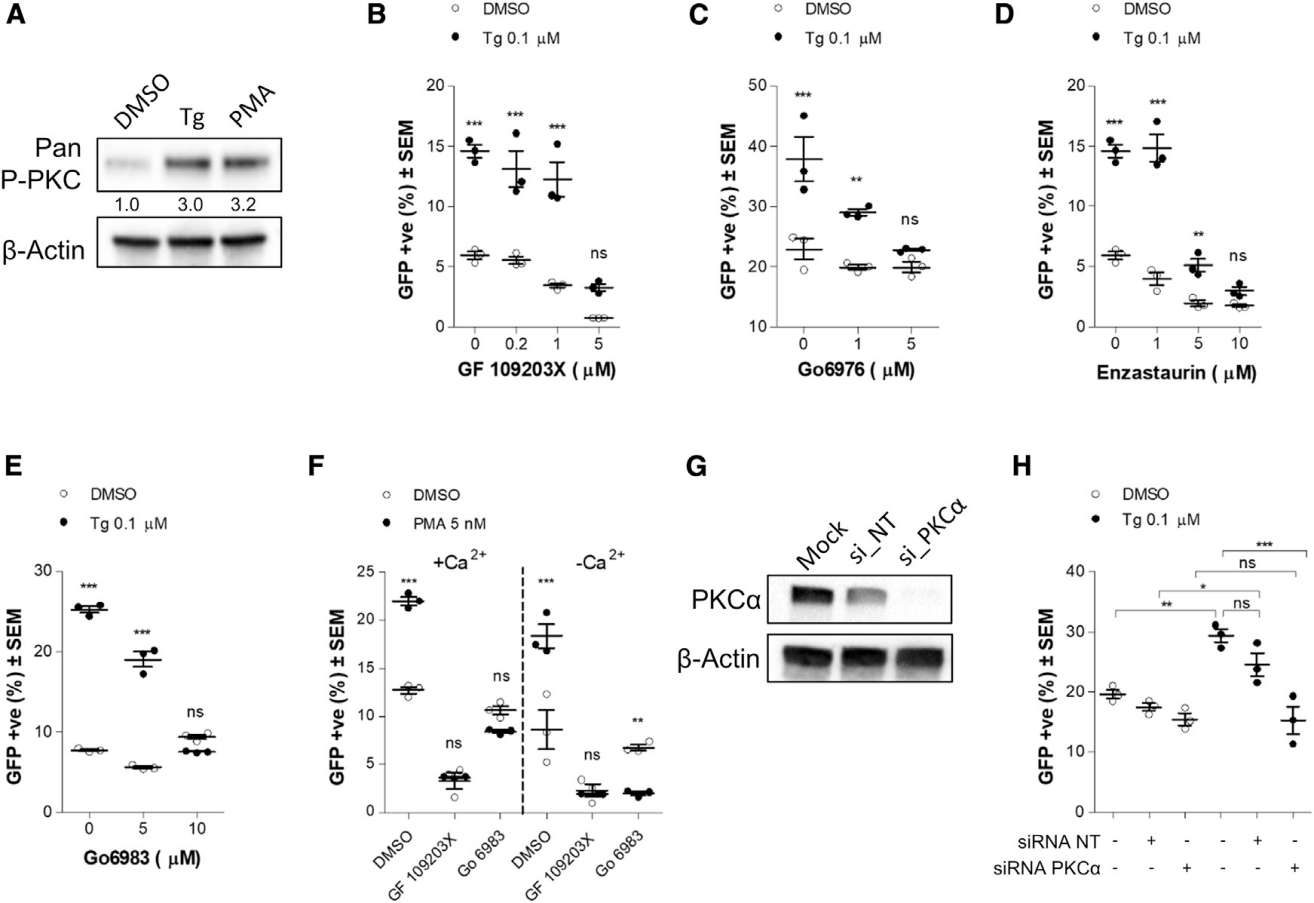
PKC Activation by Thapsigargin Mediates Enhancement of EnAd Activity (A) Western blot of phosphorylated PKC in DLD-1 cells after 30 min of stimulation with Tg, PMA (5 nM), or vehicle control DMSO. (B–F) DLD-1 cells were infected with EnAd-SA-GFP prior to exposure to Tg: (B) GF 109203X; (C) Gö 6976; (D) Enzastaurin; (E) Gö 6983 and (F) various PKC inhibitors. Treatments were performed in normal DMEM (supplemented with 2% FCS) or Ca^2+^-free DMEM (supplemented with 2% dialyzed Ca^2+^-free FCS) as indicated. Cells were imaged at 24 h postinfection using Celigo. (G) Western blot of PKCα protein levels 48 h after transfection of DLD-1 cells with either siRNAs for the targeted knockdown of PKCα or nontarget (NT) control siRNA. (H) DLD-1 cells were transfected with siRNA targeting PKCα or NT control siRNA. At 48 h post-transfection, cells were infected with EnAd-SA-GFP (100 VPC) and subsequently treated with either Tg or vehicle control DMSO. At 24 h postinfection, the proportion of GFP-positive cells was measured by Celigo. Significance was assessed by two-way ANOVA (B–F) or one-way ANOVA (H). *p ≤ 0.05; **p ≤ 0.01; ***p ≤ 0.001; ns, nonsignificant.

The PKC family of kinases includes important cellular signal transducers. Of the numerous PKC isoforms, the α, βI, βII, and γ isoforms require Ca^2+^ for activation, whereas isoforms δ, ε, η, μ, and θ are not sensitive to Ca^2+^.^42^^,^^43^ With the use of an antibody specific to the phosphorylated forms of isoforms α, βI, βII, δ, ε, η, and θ, we observed an increase in levels of phosphorylated PKC following Tg treatment of DLD-1 cells for 30 min (Figure 6A). Phorbol myristate acetate (PMA) was used as a positive control for PKC activation. Additional antibodies with specificity for isoforms α/βII, δ, μ, and θ were also used (Figure S3). These revealed a small Tg-induced increase in PKC α/βII phosphorylation and no increase in levels of phosphorylation of the other PKC isoforms. These results support the hypothesis that PKC activation is induced by Tg-mediated calcium influx, as the α/β isoforms are activated in response to calcium.

We tested whether increased PKC activity was responsible for enhancement of EnAd transgene expression by using small molecule inhibitors of PKC in the treatment media after infection. PKC blockade by the inhibitors Enzastaurin, GF 109203X, Gö 6976, and Gö 6983 abrogated the enhancing effect of Tg treatment on late transgene expression by EnAd-SA-GFP (Figures 6B–6E), whereas activation of PKC by treatment of cells with PMA resulted in a significant increase in GFP expression. Of the inhibitors, GF 109203X, Gö 6976, and Enzastaurin were selective toward Ca^2+^-responsive PKC isoforms, whereas Gö 6983 has a broad spectrum of inhibition. Virus-driven transgene expression by PMA treatment was not dependent on extracellular calcium and was reversed or reduced by inclusion of PKC inhibitors in the treatment medium (Figure 6F). These results are further supported by our observation that PMA treatment of cells enhanced E1A expression levels from EnAd (Figure S1B) in both the presence or absence of extracellular calcium. The cytotoxicity of these chemical treatments was assessed by MTS, with no significant decrease in cell viability compared to vehicle control-treated cells (Figure S4).

To validate further the role of PKC, we performed a targeted knockdown of PKCα using small interfering RNAs (siRNAs). DLD-1 cells were transfected with an siRNA with sequence complementarity specifically to the PKCα isoform or serving as a negative control with a nontarget siRNA (siRNA NT). At 48 h post-transfection, western blotting with an antibody specific to the PKCα isoform revealed a highly effective knockdown (Figure 6G). When cells were infected at 48 h post-transfection with siRNAs and subsequently treated with Tg or vehicle control DMSO, we found that knockdown of PKCα abrogated the effect of Tg on virus activity. In untransfected cells or cells transfected with siRNA NT, Tg treatment significantly enhanced the proportion of GFP-positive cells. In cells transfected with PKCα-targeted siRNA, no enhancement was observed (Figure 6H).

## Discussion

Adenoviruses are one of the most widely used vectors for oncolytic virotherapy.^23^ Although little is known about the role of ER stress and the UPR in the tumor microenvironment on adenoviral infection, previous studies indicated that chemical induction of ER stress and the UPR boosts Ad5 oncolysis through an unknown mechanism.^18^ We sought to investigate whether such an effect might be applicable to other oncolytic adenoviruses, particularly the chimeric group B oncolytic adenovirus EnAd, a promising candidate in clinical trials (E. Calvo et al., 2014, J. Clin. Oncol., abstract).^30^ Our initial studies using a reporter EnAd virus indicated that induction of ER stress by treating ovarian or colorectal cancer cells with the ER stress inducers Tg or Im did indeed lead to a substantial enhancement of virus activity in the cell lines used (Figures 1A and 1B). Whereas Tg treatment sped up target cell killing (Figure 2) and viral mRNA and protein levels (Figure 3) compared to infected but untreated controls, the effect was independent of the UPR (Figure 4). More in-depth studies revealed that the enhancing effect was due to Ca^2+^ influx rather that ER stress induction. The failure of IRE1 inhibition to ablate the increases in EnAd E1A transcript levels, the XBP-1 splicing patterns generated by the different treatments, and the inability of SubAB or Tm to enhance Ad5 E1A-Luc or virus reporter protein expression together demonstrated that induction of the UPR was not responsible for the enhancement to virus activity produced by Tg and Im treatments (Figure 4). Chemical inhibition of the IRE1 and PERK arms of the UPR also failed to block the enhancing effect of Tg treatment on activity of EnAd-SA-GFP (Figure S2). Interestingly, the addition of Tg to cells treated with the ATF6 inhibitor did not rescue late transgene expression compared to cells without ATF6 inhibition, although we cannot rule out that the trapping and clustering of ATF6α complexes in the ER by Ceapin-A7^37^ may also have downstream negative consequences on viral protein localization that counteract any potential increases in transgene level driven by Tg treatment. Instead, we found that enhancement is mediated by an influx of extracellular Ca^2+^ (Figure 5), triggering PKC activation (Figure 6).

It is unclear exactly which stage of the virus replication cycle is involved with Tg- or Im-induced upregulation. As EnAd and Ad11p differ in E2B, E3, and E4ORF4, but still exhibit similar responses to Tg and Im treatment, it is unlikely that these three genes are involved. Here, we can exclude attachment and entry, since the treatments were first added at 2 h p.i. when the majority of viral particles would have already entered the cell.^38^ Although we cannot exclude the possibility that the treatments aid viral endosome escape and delivery of viral genomes to the nucleus, previous work by Greber et al.^38^ suggests that calcium depletion in the ER/nuclear lumen by Tg or Im treatment does not affect trafficking of the virus to the nucleus. However, depletion of Ca^2+^ stores by Im treatment did block delivery of viral genomes to the nucleus once the virus particles had reached the nuclear envelope. In this study, we observe an increase in virus activity following Im treatment, suggesting that delivery of the viral genomes to the nucleus has already taken place by the time the treatments were added at 120 min p.i. (Figures 1 and 2).

We found that E1A and E2B were upregulated by Tg treatment at both the mRNA and protein levels (Figures 3A–3D). It is important to note that due to the central role the E1A protein plays in driving the expression of the other viral genes, including other early genes, elevated levels of E1A may be sufficient to drive an accelerated onset of viral replication and potentiated virus activity. The enhanced E1A mRNA levels were seen as early as 6 h p.i. (Figure 3) prior to the onset of viral genome replication, thereby excluding the possibility that the enhancing effect was acting directly on DNA replication to increase genome copy number and raise transcript levels. Subsequently, higher levels of E2B mRNA levels further support our conclusion that Tg-mediated enhancement occurs at the E1A transcriptional level or earlier.

Since both Tg and Im are known to disrupt Ca^2+^ flux, we postulated that changes in Ca^2+^ flux brought about by the treatments might account for the observed effects on virus activity. We found that Tg and Im treatments led to spikes in cytosolic Ca^2+^ levels by facilitating Ca^2+^ influx from outside the cell (Figure 5A). Indeed, influx of extracellular Ca^2+^ was critical to the enhancement in virus activity observed upon treatment with Tg or Im, since the removal of extracellular Ca^2+^ ablated the effect (Figure 5B); this effect was dose dependent on extracellular Ca^2+^ (Figures 5C and 5D). So too did the blockade of plasma membrane CRAC channels (Figures 5E and 5F), which mediate store-operated Ca^2+^ entry and are activated when the ER is depleted of Ca^2+^.^39^ Extracellular Ca^2+^ was also required for the Tg-induced increase in levels of EnAd FLAG-E1A protein (Figure 5G) and Ad5 E1A protein and mRNA (Figure 5H). Taken together, these results suggest that Tg or Im treatment depletes the intracellular stores of Ca^2+^, triggering the influx of extracellular Ca^2+^ through the CRAC channels and subsequently enhancing virus E1A transcription and virus replication.

Calcium has been shown to be important during entry of adenoviral particles,^38^ and some viruses are known to express viroporins to facilitate calcium influx for efficient viral exit and spread.^44^^,^^45^ A previous study by Gros et al.^46^ identified an Ad5-based mutant, AdT1, containing a mutation in E3-19K (E3/19K-445A) that redirected the resulting truncated protein to the plasma membrane, improving extracellular calcium influx and consequently increasing viral spread. The authors speculate that the observed improved viral spread and plaque formation are a result of better membrane permeabilization or virion release, rather than affecting earlier stages of the virus replication cycle. Indeed, they found that transcript and protein levels remained unaffected in the presence of truncated E3-19K compared to wild-type E3-19K, whereas in our study, we found major differences in transgene expression directly after infection and in the late phase of virus protein expression. Therefore, we conclude that the effect on transgene expression of UPR-modulating drug treatment in Figures 1A and 1B acts on an earlier stage in the virus replication cycle than the E3-19K truncation discovered by Gros et al.^46^

On a mechanistic level, we have shown that the influx of extracellular Ca^2+^ into the cytosol, induced by treatment with Tg or Im, results in increased activation of PKC. The α/β PKC isoforms are known to be activated by increases in Ca^2+^ concentrations.^42^^,^^43^ We observed increased phosphorylation of these isoforms upon Tg treatment via western blot. PKC inhibition blocked Tg- and Im-mediated enhancement of virus activity (Figures 6B–6E), whereas PKC activation by PMA increased virus activity (Figure 6F). To rule out the possibility that PKC activation by PMA was simply bringing about Ca^2+^ influx into the cell and mimicking the effect of Tg and Im treatments, we performed the same experiment in the absence of extracellular Ca^2+^ and found that the enhancing effect was still present (Figures 6E and 6F), suggesting that the effect was mediated through PKC signaling. Knockdown of PKCα abrogates any enhancement that Tg confers on late transgene expression (Figure 6H). Taken together, our results indicate that the influx of Ca^2+^ induced by Tg and Im treatments activates PKC, which in turn, enhances virus activity through increasing E1A levels either by the direct action of PKC or its downstream signaling pathway.

The PKC family of serine/threonine kinases regulates many cell functions that are deregulated in cancers, including differentiation, cell growth, survival, and cell death.^47^ PKC has numerous downstream cellular targets, one of which is the transcriptional coactivator p300. Like PKC, p300 has been implicated in the expression of genes involved in a diverse number of biological functions.^48^ PKC is reported to phosphorylate p300 at serine 89, having an inhibitory effect on its function.^49^ p300 also interacts with the adenoviral E1A protein (indeed, p300 was first discovered through this interaction), and E1A is thought to repress p300 activity by sequestration through binding. Since the E1A-directed repression of p300 is clearly important to viral infection, it may be that PKC activation by Tg treatment aids E1A in this repression of p300, thereby potentiating virus activity. However, more work is needed in order to elucidate any such complex signaling interaction. This is especially true when considering group B adenovirus biology, since the interactions described above were predominantly discovered by research using Ad5, and the amino acid identity of the Ad5 and Ad11 E1A protein is as low as 35%.^50^ PKCα signaling has previously been implicated in adenoviral infections. Yousuf et al.^51^ reported that inhibition of PKC reduced E1A expression. This agrees with our data, suggesting that PKC signaling may upregulate E1A expression, although the authors conclude that the upregulation of E1A levels in their model is due to enhanced viral entry, which as we discuss above, is not the mechanism described in our study.

The responses of Ad5 and EnAd/Ad11p to Tg and Im are strikingly different. Although it is difficult to speculate about the reasons for such differences, some insight can be gained from the consideration of their distinct replicative kinetics. The prevailing view of adenovirus DNA replication is based predominantly on studies using the Ad5 serotype. Initiation of DNA replication is dependent on the expression of the E2 transcription unit, consisting of viral genes E2A and E2B, and a further three host factors.^52^ The E2 transcription unit is initially controlled by the E2 early promoter, but during the intermediate stages of infection, control shifts to the E2 late promoter. It is the E2 early promoter that is regulated by the E1A protein.^53^ Thus, for E1A to drive accelerated onset of viral replication at early stages, E1A must represent a bottleneck to E2 early promoter-driven expression of the E2 transcription unit. During EnAd and Ad11p infection, this must be the case since elevated levels of E1A at early time points drive an upregulation of E2B expression and its corollary, enhanced genome replication. However, this is not the case with Ad5, as genome replication is not enhanced despite increased levels of E1A expression (Figure 3D). Indeed, previous reports suggest that adenovirus replication is not necessarily linearly dependent on enhanced E1A levels.^54^ Here, the authors found that high levels of E1A expression driven by the strong and constitutive CMV promoter did not always translate to increased replication levels of Ad5 mutant viruses. The ability of raised E1A levels to drive higher rates of Ad5 replication was dependent on the cell type and Ad5 variant used. It is also well established that tumor suppressor signaling pathways, such as the p53 pathway, which are often deregulated in cancer cells, play an important role in regulating viral replication. Since viral replication is influenced by a number of such variables, it follows that the sensitivity of replication to E1A levels will not be uniform, and should depend on both the cell type and the virus used. Because Ad11p and Ad5 are relatively nonhomologous (54% homology), differences in their biology should be expected and extreme caution should be used when extrapolating about one virus from knowledge of the biology of the other. These differences could be studied with a wider panel of adenoviruses, although given the large differences already apparent between only two subtypes, this would likely be a mammoth task that would warrant a stand-alone study. Clearly, more detailed investigations into the reasons why elevated E1A levels are sufficient to drive enhanced replication of Ad11p, but not Ad5, in these cell lines are needed before any precise explanation can be described.

We do not currently have a complete understanding of the many interactions between adenovirus and its host cell; this is particularly true in the case of the adenovirus subtypes, which are most genetically distinct compared to Ad5. Further understanding of these interactions and the ways in which oncolytic adenoviruses behave and replicate, especially in the conditions found within the tumor microenvironment, may be key to their clinical development. Although clinical trials and preclinical data so far have generated encouraging results,^55^^,^^56^ it is clear that novel approaches to maximize their therapeutic effect are needed. These results, which indicate a link between PKC signaling and the E1A protein and describe a substantial enhancement to virus activity through combination with Ca^2+^ flux-altering drugs, make an important contribution in this regard.

## Materials and Methods

### Mammalian Cell Culture

Human colorectal carcinoma cells (DLD-1), human lung carcinoma (A549), human ovarian carcinoma cells (SK-OV-3), and HEK293 were cultured in DMEM, supplemented with 10% fetal calf serum (FCS; herein known as complete culture medium) at 37°C and 5% CO_2_. All cell lines were obtained from ATCC and routinely tested for mycoplasma.

### Viruses and Virus Engineering

The genome of EnAd was modified using the parental vector ColoAd2.4 as previously described.^33^ Ad5-E1A-Luc was constructed as previously described.^35^ For Ad5-CMV-GFP, a DNA fragment encoding the CMV EGFP expression cassette was amplified by PCR using plasmid template OG4728 (Oxford Genetics, UK) with forward primers 5′-CATCTTATTCCCTTTAACTAATAAAAAAAAATAATAAAGCATCACTTACTTAAAATCAGTTAGCAAATTTATGCATGTCGACAGTAATCAATTACGGGG-3′ and reverse primers 5′-GAAATTTGCTAACTGATTTTAAGTAAGTGATGCTTTATTATTTTTTTTTATTAGTTAAAGGGAATAAGATCGCGACCTAGGATAGCTGACGACTACCACATTTGTAGAGGTTTTACTTGCTTTAAAAAACCTCCCACACCTCC-3′ and cloned into a *SalI/AvrI*I-linearized Ad5 E3-deleted shuttle plasmid (Oxford Genetics, UK) by the Gibson assembly method (NEB) to generate shuttle plasmid pAd5-E3-deleted-CMV-EGFP. A DNA insert encoding the CMV EGFP expression cassette with flanking Ad5 DNA sequences was excised using *SfiI* from pAd5-E3-deleted-CMV-EGFP and assembled into *SbfI*-linearized pSU143, a plasmid encoding a full-length wild-type Ad5 genome, to generate pSU164 (pAd5-E3-deleted-CMV-EGFP). Recombinant virus was recovered from the plasmids as follows. The viral genome plasmids were linearized by digestion with *AscI* for EnAd and *SwaI* for Ad5. Linearized fragments were precipitated using 0.6 volumes isopropanol and centrifuging for 30 min at 4°C. Fragments were resuspended in double-distilled H_2_O (ddH_2_O) and 5 μg DNA was transfected into 1 × 10^6^ HEK293A cells in a T25 flask using Lipofectamine 2000. Cells were left until plaques were visible. Supernatant was collected, and viruses were plaque purified and tested for transgene expression. Viruses were selected for purification by cesium chloride banding, as described in Tedcastle et al.^57^ Viral stocks were quantified by PicoGreen (Quant-iT PicoGreen dsDNA Assay Kit, Thermo Fisher, UK, #P11496) to calculate the number of viral genomes per milliliter as an estimate of viral particles.

### Infection Studies

Infections were performed in complete culture medium for 2 hours at 37°C before changing the medium for fresh culture medium or treatment medium as indicated, supplemented with 2% FCS. Virus dose was 100 virus particles per cell (VPC) unless otherwise stated. Cells were incubated at 37°C for the indicated number of days before harvesting and analysis.

### qPCR

Viral genomes were measured by qPCR using primers and probes specific for the hexon or fiber genes for EnAd/Ad11p and Ad5, respectively. Genomic DNA was extracted from harvested cells using the PureLink Genomic DNA Purification Kit (Life Technologies, UK, #K182001). Extracted DNA was diluted 1,000-fold and genomes quantified in a 20 μL qPCR reaction consisting of two times qPCRBIO Probe Mix Hi-Rox (PCR Biosystems, UK) and 10 μM each of forward primer, reverse primer, and probe. Cycling conditions were as follows: one cycle at 95°C for 2 min, followed by 40 cycles at 95°C for 5 s and 60°C for 30 s. Cycle threshold (C_T_) values from known quantities of virus particles were used to calculate a standard curve: EnAd/Ad11p forward primer: 5′-TACATGCACATCGCCGGA-3′, EnAd/Ad11p reverse primer: 5′-CGGGCGAACTGCACC-3′; EnAd/Ad11p probe: 5′-[6FAM]-CCGGACTCAGGTACTCCGAAGCATCCT-[TAM]-3′, Ad5 forward primer: 5′-TGGCTGTTAAAGGCAGTTTGG-3′, Ad5 reverse primer: 5′-GCACTCCATTTTCGTCAAATCTT-3′, and Ad5 fiber probe: 5′-[6FAM]-TCCAATATCTGGAACAGTTCAAAGTGCTCATCT-[TAM]-3′.

### qRT-PCR

EnAd E1A and E2B expression was measured by qRT-PCR. Total RNA was extracted using the RNeasy Mini Kit (QIAGEN, UK, #74104) with on-column DNA digestion. cDNA was generated using the QuantiTect Reverse Transcription Kit (QIAGEN, UK, #205310). mRNA copies were quantified in a 20-μL qPCR reaction consisting of two times qPCRBIO Probe Mix Hi-Rox (PCR Biosystems, UK) and 10 μM each of forward primer, reverse primer, and probe (E1A forward: 5′-CCATCTCCTGATTCTACTACC-3′, reverse: 5′-CCGTGTACTCAAGTCCAA-3′, probe: 5′-TAAGCCTGGGAAACGTCCAGCAGT-3′; E2B forward: 5′-CTCTTCAATGATGTTACTTTCG-3′, reverse: 5′-GTAGCGAAGCGTGAGTAAG-3′, probe: 5′-AGGCTCCCTGTTCCCAGAGTTGGA-3′). Sequences are given as 5′–3′. Probes are tagged with 6FAM at the 5′ end and BHQ1 at the 3′ end. Cycling conditions were as follows: one cycle at 95°C for 2 min, followed by 40 cycles at 95°C for 5 s and 60°C for 30 s. C_T_ values from known copy numbers of each gene were used to calculate a standard curve.

### Immunoblotting

Protein expression in infected cells was analyzed by immunoblotting. Infected cells were harvested by removing the supernatant from cell cultures and rinsing gently with PBS. Cells were lysed by adding Pierce radioimmunoprecipitation assay (RIPA) buffer, supplemented with one time protease inhibitor, directly to the cell monolayer and incubating at room temperature for 5 min. Lysates were scraped and transferred into 1.5 mL Eppendorf tubes and incubated with 2.5 U Benzonase for 30 min at room temperature. Lysate concentrations were measured by the QuantiPro BCA Assay Kit (QPBCA-1KT; Sigma-Aldrich, UK). Samples containing 30 μg of each protein lysate in one time Laemmli sample buffer were heated at 95°C for 5 min. Proteins were separated on a 4%–20% Mini-PROTEAN TGX Precast Protein Gel (Bio-Rad, UK) and transferred onto a 0.2-μm nitrocellulose membrane using the wet blot method. Membranes were incubated with SuperSignal West Dura Extended Duration Substrate (Thermo Fisher, UK, #34075) and bands detected using the Gel Doc system (Bio-Rad). Quantification of blots was performed by densitometry using Image Lab software. Intensity volume of bands was normalized to the relevant loading control (β-actin) band. Normalized intensity values were divided by the value of the untreated control band to give relative fold-change values. FLAG-tag was detected with a Direct-Blot horseradish peroxidase (HRP) anti-DYKDDDDK tag (BioLegend, UK, #637312). β-Actin was detected using monoclonal anti-β-actin-peroxidase antibody (Sigma-Aldrich, UK, #A3854). The following PKC-specific antibodies were purchased from Cell Signaling Technologies, UK: phospho-PKC (pan) (βII Ser660) #9371, phospho-PKCα/βII (Thr638/641) #9375, phospho-protein kinase D (PKD)/PKCμ (Ser916) #2051, PKD/PKCμ (D4J1N) #90039, phospho-PKCδ (Thr505) #9374, phospho-PKCδ/θ (Ser643/676) #9376, phospho-PKCθ (Thr538) #9377, phospho-PKCζ/λ (Thr410/403) #9378. All PKC antibodies were detected with secondary antibody anti-rabbit immunoglobulin G (IgG) HRP linked (Cell Signaling Technologies, UK, #7074).

### Ca^2+^ Flux Measurement

The fluo-8 Ca^2+^ assay (Abcam, UK, #ab142773) was used to measure intracellular cytosolic Ca^2+^ levels following the manufacturer’s protocol. In short, the dye was added to cells and incubated for 30 min at 37°C and for a further 30 minutes at room temperature. After dye loading, medium was changed for Ca^2+^-free medium, supplemented with 2% dialyzed Ca^2+^-free FCS. Fluorescence measurements were taken once per second using a POLARstar Omega plate reader spectrophotometer (BMG Labtech) at excitation/emission of 490/525 nm. At 5 min, drug treatments were injected to a final concentration on cells, as indicated, after a further 5-min calcium was added back to the indicated final concentration

### Infectious Virus Particle Quantification

Quantification of infectious virus particles was done by the 50% tissue culture infectious dose (TCID_50_) method. Cells were seeded at 50,000 cells per well in a 24-well plate and incubated overnight to adhere. Cells were then infected in triplicate and where indicated, were subsequently exposed to drug treatments. At the indicated time points, cells were scraped, and the sample was stored at −20°C until analysis. Samples were subjected to three rounds of freeze thawing to encourage cell lysis and release of cell-associated virus particles. A549 cells were seeded at 20,000 cells per well in 96-well plates in complete culture medium. The following day, 1:10 serial virus dilutions were performed in DMEM, supplemented with 2% fetal bovine serum (FBS), and used to infect the A549 cells. Cells where incubated for 5 days and then scored for the presence of viral plaques.

### Chemicals

Tg (#T9033-1MG), Im (#I3909-1ML), Tm (#T7765-1MG), 4μ8C (#SML0949-5MG), and PMA (P1585-1MG) were all purchased from Sigma-Aldrich, UK. SubAB was purified from recombinant *Escherichia coli*, as described previously.^58^ BTP2 (#sc-221441) was from Santa Cruz Biotechnology (UK). BAPTA was from Life Technologies (UK, #B1205). Gö 6976 (#2253/1), Gö 6983 (#2285/1), Enzastaurin (#5994/10), and GF 109203X (#0741/1) were purchased from Bio-Techne R&D Systems (UK).

### xCELLigence Studies

The xCELLigence RTCA DP instrument (Roche) was used for the real-time monitoring of cell growth. Cells were seeded at 20,000 cells per well in E-Plate 16 plates (ACEA Biosciences, UK). Impedance measurements for each well were then taken automatically every 10 min and expressed as a CI.

### XBP-1 mRNA Analysis

Cells were seeded in microplate wells and subsequently treated as indicated. At the indicated time points, cells were washed in PBS, and RNA was harvested using the RNeasy Mini Kit (QIAGEN, UK, #74104). RNA content was measured on a NanoDrop Microvolume Spectrophotometer (Thermo Fisher, UK), and 800 ng of RNA was reverse transcribed into cDNA using the QuantiTect Reverse Transcription Kit (QIAGEN, UK, #205310) with random primers. From the cDNA, the XBP-1 fragments were amplified using primers 5′-TTACGAGAGAAAACTCATGGCC-3′ and 5′-GGGTCCAAGTTGTCCAGAATGC-3′.^59^ The PCR product was resolved on a 2.5% agarose gel revealing amplicons for the XBP-1 full-length product (289 bp) and spliced product (263 bp).

### Analysis of Viral GFP Expression by the Celigo Image Cytometer

Cells were infected with the indicated virus and dose as described. At the indicated time points p.i., cells were imaged using the Celigo image cytometer (Nexcelom Biosciences, UK). Images were taken in both brightfield and fluorescent channel. Analysis was carried out by Celigo software using the confluence ratio program. This program measures the surface area of the cell monolayer in the brightfield channel and the surface area of the fluorescent cells in the fluorescent channel. The surface area in the fluorescent channel is then divided by that of the brightfield channel to yield a confluence ratio score. This ratio was then converted to a percentage.

### Luciferase Reporter Assay

Cells were plated in white 96-well plates and the following day, infected with Ad5-E1A-Luc virus (MOI 3); 2 hours after infection, infectious media were replaced with treatment media. Twelve hours after initial infection, cells were lysed in passive lysis buffer (Promega, UK, #E1941) and underwent three rounds of freeze thaw to encourage cell lysis. Cell debris was removed by centrifugation in a v-bottom plate. Luciferase activity was measured using the Promega luciferase assay system (Promega, UK, #E1500), following the manufacturer’s instructions, using a POLARstar Omega plate reader spectrophotometer (BMG Labtech).

### siRNA Knockdown Experiments

Silencer select siRNAs were purchased from Thermo Fisher, UK. siRNAs targeting the PKCα isoform (Assay ID: s11094) or a nontarget negative control siRNA (Negative Control No. 2, #4390846) were transfected into DLD-1 cells using Lipofectamine 2000 transfection reagent (Invitrogen, UK), following the manufacturer’s instructions. Two days after transfection, cells were harvested for immunoblotting or infected with EnAd-SA-GFP.

### MTS Assay

Cell viability was measured using the CellTiter 96 Aqueous One Solution Cell Proliferation Assay (Promega), according to the manufacturer’s instructions.

### Statistical Analysis

When comparing two datasets, a Student’s two-tailed t test was used. Where more than two groups were being compared, one-way analysis of variance (ANOVA) test was used with Tukey’s post hoc analysis. For grouped datasets, two-way ANOVA test was used with Bonferroni post hoc analysis. All data are presented alongside bars indicating ± standard error in the mean (SEM). The significant levels used were *p = 0.01–0.05, **p = 0.001–0.01, and ***p ≤ 0.001.

## Author Contributions

Conceptualization, W.K.T., E.J.J., J.C., B.C., L.W.S., and J.L.-R.; Methodology, W.K.T., E.J.J., J.C., W.S., and J.L.-R.; Investigation, W.K.T.; Resources, J.C., J.C.P., A.W.P., W.S., and R.C.; Writing – Original Draft, W.K.T.; Writing – Review & Editing, L.W.S. and J.L.-R.; Supervision, L.W.S. and J.L.-R.

## Conflicts of interest

L.W.S. and B.C. own equity or share options in PsiOxus or are employed by the company.

## Supplemental Information

Document S1. Figures S1–S4

Document S2. Article plus Supplemental Information
