# Supplementary material for: Calcium Influx Caused by ER Stress Inducers Enhances Oncolytic Adenovirus Enadenotucirev Replication and Killing through PKCα Activation
Source: Mol Ther Oncolytics. 2019 Sep 28;15:117–30. doi: 10.1016/j.omto.2019.09.003 (PMC6931121; doi:10.1016/j.omto.2019.09.003)
Supplement: Document S1. Figures S1–S4 [file mmc1.pdf]

OMTO, Volume *15*

Supplemental Information

Calcium Influx Caused by ER Stress Inducers Enhances Oncolytic Adenovirus Enadenotucirev Replication and Killing through PKCα Activation

William K. Taverner, Egon J. Jacobus, John Christianson, Brian Champion, Adrienne W. Paton, James C. Paton, Weiheng Su, Ryan Cawood, Len W. Seymour, and Janet Lei-Rossmann

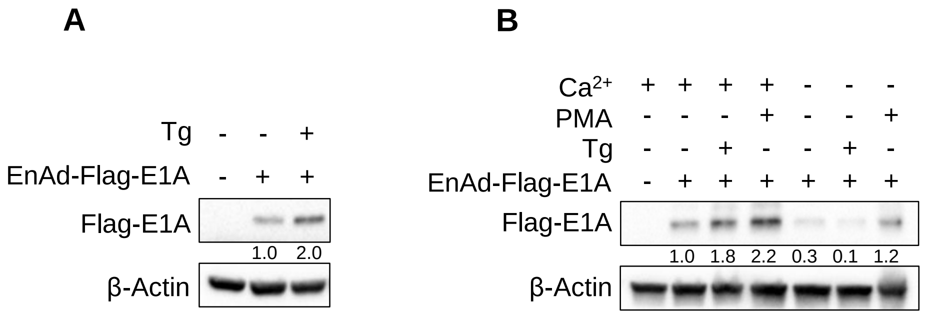

Figure S1. Western blot analysis of EnAd FLAG-E1A protein levels in DLD-1 cells 12 h post-infection. **A)** Cells were infected at an MOI of 3 and subsequently exposed to Tg (0.1 µM). **B)** Cells were infected at an MOI of 3 and subsequently exposed to Tg (0.1 µM) or PMA (5 nM) in the presence or absence of extracellular calcium. At 12 h post-infection, cell lysates were probed for FLAG-E1A expression using an anti-FLAG antibody via western blot.

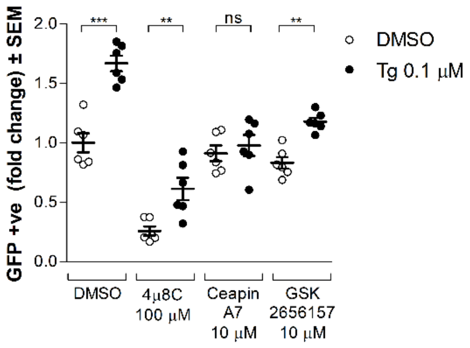

Figure S2. Impact of UPR signalling inhibitors on the effect of thapsigargin treatment on virus activity. DLD-1 cells were infected with EnAd-SA-GFP (100 VPC) and subsequently exposed to treatments at indicated concentrations. At 24 h post-infection, the proportion of GFP-positive cells was analysed by Celigo. Significance was assessed by one-way ANOVA. **, $p \leq 0.01$; ***, $p \leq 0.001$; ns, not significant. Error bars indicate ± SEM.

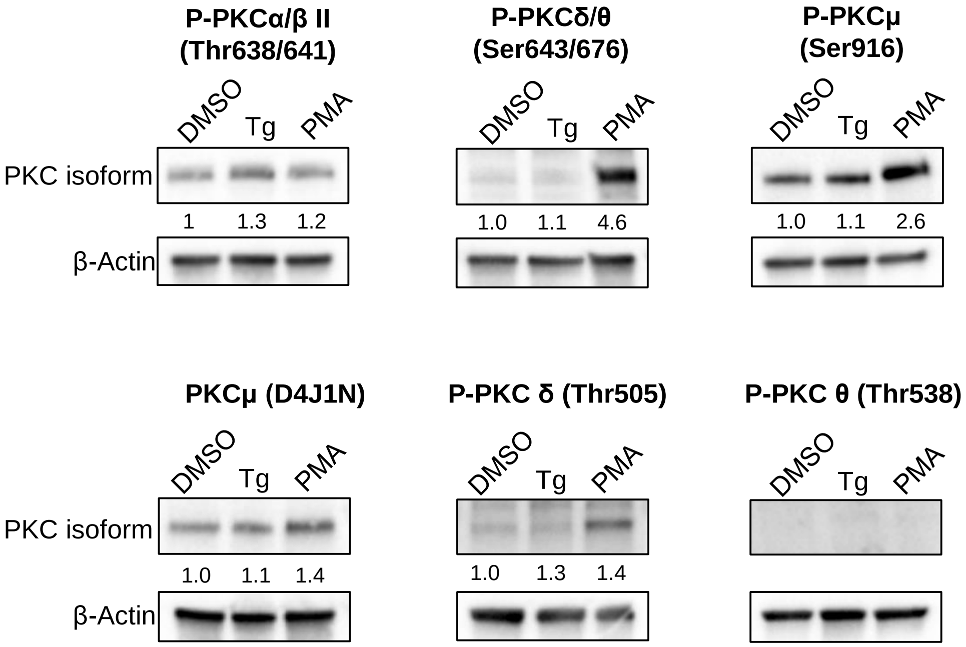

Figure S3. PKC and phospho-PKC isoform levels. Western blot of DLD-1 cells exposed to treatment with 0.1 μM Tg or PMA (5 nM) for 30 mins. Numbers indicate the normalised fold-change in band volume intensity relative to the DMSO-treated sample.

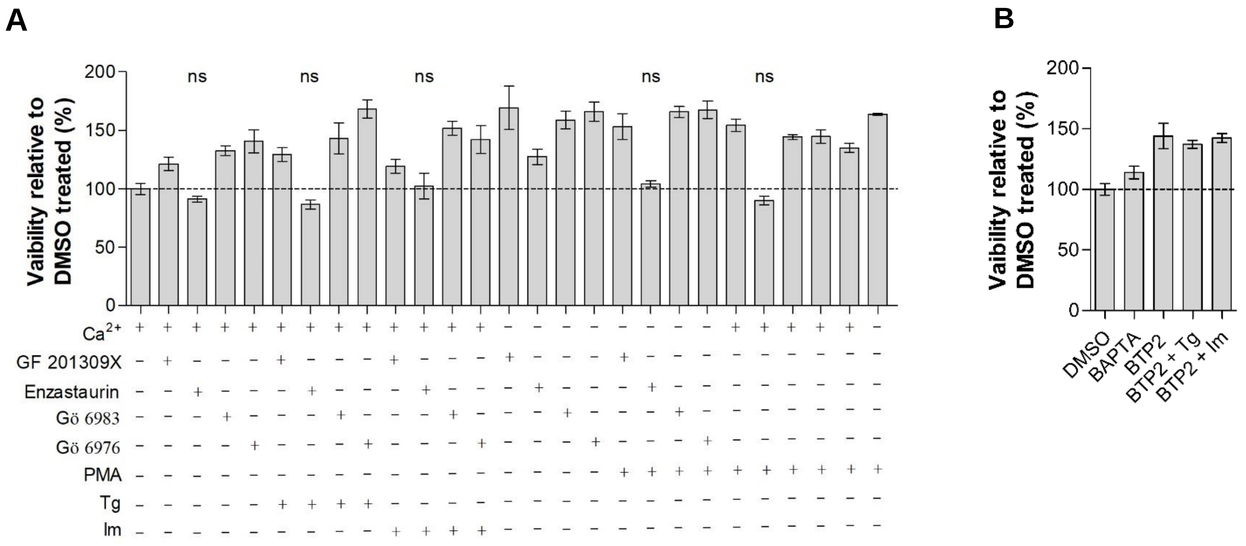

Figure S4. Cytotoxicity of drugs used in this study. A, B) DLD-1 cells were treated at the maximum concentrations used in this study for 24 h before cytotoxicity was assessed via MTS. The mean of 4 replicates is plotted with error bars indicating ± SEM. Significance was assessed by two-way ANOVA. Ns: not significant.
